# Age-related changes in neural oscillations vary as a function of brain region and frequency band

**DOI:** 10.3389/fnagi.2025.1488811

**Published:** 2025-02-18

**Authors:** Jinhan Park, Rachel L. M. Ho, Wei-en Wang, Shannon Y. Chiu, Young Seon Shin, Stephen A. Coombes

**Affiliations:** ^1^Laboratory for Rehabilitation Neuroscience, Department of Applied Physiology and Kinesiology, University of Florida, Gainesville, FL, United States; ^2^Department of Neurology, Mayo Clinic, Scottsdale, AZ, United States; ^3^Department of Biomedical Engineering, University of Florida, Gainesville, FL, United States

**Keywords:** EEG, alpha power, beta power, age, aperiodic components, periodic components

## Abstract

Advanced aging is associated with robust changes in neural activity. In addition to the well-established age-related slowing of the peak alpha frequency, there is a growing body of evidence showing that older age is also associated with changes in alpha power and beta power. Despite the important progress that has been made, the interacting effects of age and frequency band have not been directly tested in sensor and source space while controlling for aperiodic components. In the current study we address these limitations. We recruited 54 healthy younger and older adults and measured neural oscillations using a high-density electroencephalogram (EEG) system during resting-state with eyes closed. After preprocessing the EEG data and controlling for aperiodic components, we computed alpha and beta power in both sensor and source space. Permutation two-way ANOVAs between frequency band and age group were performed across all electrodes and across all dipoles. Our findings revealed significant interactions in sensorimotor, parietal, and occipital regions. The pattern driving the interaction varied across regions, with older age associated with a progressive decrease in alpha power and a progressive increase in beta power from parietal to sensorimotor regions. Our findings demonstrate that age-related changes in neural oscillations vary as a function of brain region and frequency band. We interpret our findings in the context of clinical and preclinical evidence of age effects on the cholinergic circuit and the Cortico-Basal Ganglia-Thalamo-Cortical (CBGTC) circuit.

## Introduction

Advanced aging is associated with robust changes in neural activity. The modulation of neural activity during resting states has been fundamental in advancing our understanding of the neural circuits that subserve age-related changes in cognitive function and motor performance (Jensen and Mazaheri, 2010; Foxe and Snyder, 2011; Klimesch, 2012; Bönstrup et al., 2015; Heinrichs-Graham et al., 2018; Rempe et al., 2022). In addition to the well-established age-related slowing of the peak alpha frequency (Scally et al., 2018; Capilla et al., 2022; Merkin et al., 2023; Park et al., 2024), there is a growing body of EEG and magnetoencephalography (MEG) evidence showing that older age is also associated with changes in alpha power (Babiloni et al., 2006; Scally et al., 2018; Jabès et al., 2021; Merkin et al., 2023; Tröndle et al., 2023; Park et al., 2024) and beta power (Vysata et al., 2012; Gómez et al., 2013; Heinrichs-Graham et al., 2018; Rempe et al., 2022). Modulation in the power of alpha oscillations is most prevalent in posterior sensors at the scalp level (Barry and De Blasio, 2017) and parietal and occipital regions at the cortical level (Babiloni et al., 2006), whereas changes in beta power have been localized to central sensors at the scalp level (Barry and De Blasio, 2017) and sensorimotor regions at the cortical level (Heinrichs-Graham et al., 2018; Rempe et al., 2022). To date, the majority of aging studies have focused on either alpha or beta power (Vysata et al., 2012; Gómez et al., 2013; Barry and De Blasio, 2017; Rempe et al., 2023), have assessed power at the scalp level (Vysata et al., 2012; Gómez et al., 2013; Barry and De Blasio, 2017; Scally et al., 2018; Jabès et al., 2021; Merkin et al., 2023), and have done so without controlling for aperiodic components (Rempe et al., 2023). As such, the interacting effects of age and frequency band have not been directly tested across the whole cortex in source space while controlling for aperiodic components. The goal in the current study is to address this gap in the literature.

Electrophysiological signals exhibit a 1/f-like distribution in frequency space, demonstrating an exponential decrease in power spectra from lower to higher frequencies. These are referred to as aperiodic components, which have long been considered background noise. However, recent evidence suggests that these aperiodic components reflect physiological processes (Manning et al., 2009; Gao et al., 2017; Donoghue et al., 2020), that can be represented by the calculation of offset and exponential slope. Offset represents a uniform shift in the entire power spectra across frequencies. An invasive EEG study using electrodes implanted in the human brain revealed that offset values are positively associated with neural population spiking in the neocortex (Manning et al., 2009). This suggests that higher offset values correspond to faster neural firing rates. Moreover, the exponential slope of the 1/f-like distribution was found to be correlated with the ratio between cortical excitatory and inhibitory activity (Gao et al., 2017). This correlation manifests as a flatter slope when the excitatory activity is higher relative to inhibitory activity, and a steeper slope when the reverse is true. Furthermore, these aperiodic components can be influenced by healthy aging. Several recent studies have found that older adults exhibit a lower offset and a flatter slope compared to young adults (Donoghue et al., 2020; Merkin et al., 2023; Tröndle et al., 2023).

Convergence on how age alters alpha and beta band power during resting states has yet to be reached. Although decreases in alpha power and increases in beta power have been reported with age (Gómez et al., 2013; Barry and De Blasio, 2017), other studies have reported opposing findings, such as an age-related increase in alpha power (Doval et al., 2023; Rempe et al., 2023) and decrease in beta power (Vysata et al., 2012). A decrease in alpha power and no change in beta power has also been found (Babiloni et al., 2006; Jabès et al., 2021). The discrepancy in findings may be related to whether aperiodic components were controlled for in the analysis. For instance, Merkin et al. (2023) compared age effects on alpha power with and without adjusting for the aperiodic components. Significant age-related differences were found only in alpha power without adjustment. However, when adjusting for the aperiodic components, significant differences emerged in beta power after averaging data across all electrodes. Properties of the aperiodic components may therefore mask age-related changes in beta power, but it is unclear if this effect is generalizable or is limited to specific regions of the cortex. The potential effect(s) of age on regional specific changes in periodic alpha and beta power has yet to be directly tested in sensor and source space.

**FIGURE 1:**
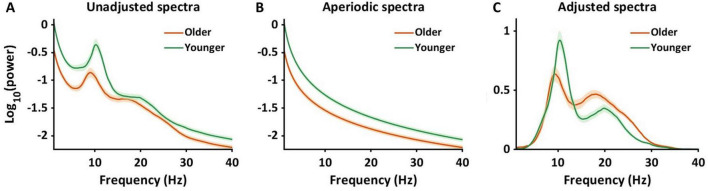
Adjusting power spectra using aperiodic components. It illustrates the changes in power spectra after the adjustment of aperiodic components in sensor space. All power spectra were log-transformed and averaged across 128 channels and individuals. **(A)** The power spectra were shown ranging from 1 to 40 Hz, unadjusted for aperiodic components. The green color represents younger adults, and the orange color represents older adults. The shaded areas indicate the standard error for power at each frequency. **(B)** The panel presents power spectra for aperiodic components for younger and older adults. **(C)** After controlling for the aperiodic power spectra, we obtained the power spectra shown in the panel. This study used the adjusted power spectra **(C)** to calculate alpha and beta power to investigate the interaction effects on periodic power between age and frequency band.

In the current study, we assessed 128-channel resting-state EEG data of cognitively healthy young and older adults. To analyze periodic alpha and beta power in sensor and source space, we controlled for aperiodic components using the Fitting Oscillations and One Over F (FOOOF) algorithm (Donoghue et al., 2020). The FOOOF algorithm parameterizes aperiodic and periodic components. To illustrate how the algorithm works, Figure 1A represents the original power spectrums from 1 to 40 Hz, averaged across all channels, for the younger (green) and older (orange) groups. Figure 1B shows the aperiodic spectra, calculated by the FOOOF algorithm from the original data. We calculated offsets and exponential slopes based on the aperiodic spectra (Figure 1B). The periodic spectrum is then shown in Figure 1C, which represents the power spectral density plot with the aperiodic component removed. This study used the adjusted power spectra (Figure 1C) to investigate the age by frequency interaction effects on periodic power using statistical analysis in sensor space and source space. We tested the hypothesis that compared to younger adults, older adults would exhibit lower offsets and flatter slopes as well as lower alpha power and higher beta power.

## Materials and methods

### Participants

We used EEG datasets from our previous study (Park et al., 2024). The subjects were twenty-eight younger adults (22 females, 6 males, 20 ± 1.7 years old) and twenty-six older adults (18 females, 8 males, 65 ± 7.9 years old). No subject reported a history of neurological disorder or head trauma. All subjects signed an informed consent form that was approved by the local Institutional Review Board (IRB) at the University of Florida (IRB201600761).

The sample size was determined through a power analysis using G*Power software (version: 3.1.9.7), with an alpha level of 0.05 and a statistical power of 0.95. Based on previous findings, which revealed a medium effect of age on alpha power adjusted by aperiodic components (Tröndle et al., 2023), we set the effect size (partial η^2^) of 0.06, indicating a medium effect. The minimum sample size was determined to be 54 subjects.

### EEG data acquisition

The ActiveTwo system, including 128 Ag-AgCl active electrodes and a 256-channel AD box (BioSemi, Amsterdam, Netherlands), was used to acquire EEG data. This system replaces the conventional ground electrode with two separate electrodes: the common mode sense and the driven right leg. These electrodes form a feedback circuit that leads the subject’s average potential as close as possible to the reference voltage in the AD box. The DC offset indirectly measures impedance tolerance and allowed us to monitor signal quality from active electrodes. The DC offsets, which are the averages of the voltages acquired between the common mode sense and each active electrode, were kept under 40 μV. The resting state EEG (rsEEG) data, sampled at a rate of 2,048 Hz, were collected for 10 min with eyes closed.

### EEG data processing

EEG data were processed using customized MATLAB scripts that leveraged the EEGLAB and Fieldtrip toolboxes. The preprocessing consisted of five steps. (1) EEG data were down sampled from 2,048 to 256 Hz. (2) The middle 8 min of EEG data were extracted. (3) Data were band-pass filtered from 1 and 100 Hz and line noise at 60 and 120 Hz was removed. (4) Using correlations between channels’ signals, bad channels were identified if the correlation was below 0.4. The bad channels’ signals were removed and interpolated based on neighboring channels. (5) The EEG data were re-referenced using the overall average of all channels. Preprocessing parameters are summarized in Supplementary Table 1.

### Independent component analysis and artifact removal

The Adaptive Mixture Independent Component Analysis (AMICA) algorithm was used to decompose independent components (ICs) from EEG data (Palmer et al., 2011). The decomposed ICs were classified as either brain signals or non-brain signals using IC label (Pion-Tonachini et al., 2019). Non-brain sources included scalp and neck muscle activity, electro-oculographic activity associated with eye blinking, saccades, and ocular motor tremor, electrocardiographic signals, and single-channel noise. ICs were automatically removed based on specific criteria (Supplementary Table 1). Following this, the EEG data were re-evaluated using the Artifact Subspace Reconstruction algorithm to discard “bad” sections of the data based on a large standard deviation in 0.5 sec windows and large amplitude EEG data within each channel. To eliminate artifactual epochs from the EEG data, we divided the data into 6-s epochs ranging from −2 to 4 s and employed the Automatic Epoch Rejection algorithm, using the thresholds of mu-volts, joint-probability, and kurtosis.

### EEG source localization

We employed the exact low resolution brain electromagnetic tomography (eLORETA; Pascual-Marqui et al., 1994) method to calculate the cortical distribution of scalp electrical potentials acquired from all 128 EEG electrodes. eLORETA is a weighted minimum norm solution (Pascual-Marqui, 2007) which estimates distributed electronic potentials from cortical dipoles based on an assumption that every dipole has a minimum energy (Pascual-Marqui et al., 1994). Prior to conducting the eLORETA analysis, we created a head model using the boundary element model (BEM). The BEM head model includes an electrode location map based on the standard 10-5 system. The electrode location map was translated onto the BEM template map, which in turn, was coregistered to the Montreal Neurological Institute (MNI) brain template. Source space was limited to 20,464 cortical region vertices which are called dipoles, used as a source model. Based on the head and source models, we conducted eLORETA and obtained power spectrums for all dipoles, ranging from 1 to 40 Hz. Power spectrums in all dipoles were computed based on Welch’s method (Welch, 1967).

### Alpha and beta power calculation

For computation of power spectrums in sensor and source space, 1–40 Hz frequency bands were used to remain consistent with previous studies (Merkin et al., 2023; Tröndle et al., 2023) and our prior work (Park et al., 2024). Since fast frequency power spectrums were not our focus, the knee point, which is observed within faster gamma frequencies was not assessed or controlled for in the current analysis. Based on the sensor and source power spectrums, we calculated aperiodic and periodic components using the FOOOF algorithm.

Prior to calculating periodic alpha and beta power, the accuracy of the model fits resulting from the FOOOF algorithm was tested using r^2^ and mean squared error between original and modeled power spectra. The r^2^ and error values were over 0.94 and less than 0.15 for both groups (Supplementary Figure 1), suggesting that the model fits accurately explained the original power spectra. Using the periodic power spectrum (Figure 1C), alpha power within the 8–13 Hz frequency band and beta power within 13.1–30 Hz frequency band was extracted and averaged for each sensor for each individual. Aperiodic components, including offset and exponential slope, were also extracted for each sensor and each individual. For the source analysis, power spectrums for all 20,464 dipoles were calculated. Similar to the sensor space analysis, the FOOOF algorithm was used to compute the periodic spectrum ranging from 1 to 40 Hz for each dipole. Average alpha and beta power were calculated for each dipole for each individual.

### Statistical analysis

Statistical analyses were conducted separately in sensor space and in source space using the same approach.

To identify brain regions that show significant interaction effects between group (younger and older) and frequency band (alpha and beta power), mixed model permutation two-way ANOVAs were conducted at each electrode and dipole. For regions with a significant interaction, we extracted and averaged (1) alpha power, (2) beta power, (3) offset, and (4) slope and conducted permutation *t*-tests to explore between group differences in each of these measures.

Cognizant that interaction effects could be driven by region specific differences in alpha and/or beta power, permutation *t*-tests were conducted at each electrode and dipole within areas showing an interaction effect to identify where alpha power was different between groups and where beta power was different between groups. For each region we extracted and averaged (1) alpha power, (2) beta power, (3) offset, and (4) slope and conducted permutation *t*-tests to explore between group differences.

Finally, a conjunction analysis was conducted within interaction regions. Electrodes and dipoles where alpha power was different between groups were given a label of 1. Electrodes and dipoles where beta power was different between groups were given a value of 2. The maps were then summed, to isolate (1) areas of the scalp and cortex where only alpha power differed between groups (i.e., a value of 1); (2) areas where only beta power differed between groups (a value of 2); and (3) areas where both alpha and beta differed between groups (a value of 3). For each region we extracted and averaged (1) alpha power, (2) beta power, (3) offset, and (4) slope. Permutation *t*-tests were then computed to compare group effects in each sub region. To determine whether aperiodic components differed across regions/electrodes identified in the conjunction analysis in source space we calculated Hedges’ gs and 95% confidence intervals for between group contrasts in both offset and slope.

All sensor and source space ANOVAs and *t*-tests were performed using a permutation method with 4,000 shuffles. Significance level was set at *p* < 0.05, and all *p*-values were adjusted by the FDR correction (Benjamini et al., 2006). All statistical analyses were conducted using customized MATLAB code.

## Results

### Electrode space: age by frequency band interaction

**FIGURE 2:**
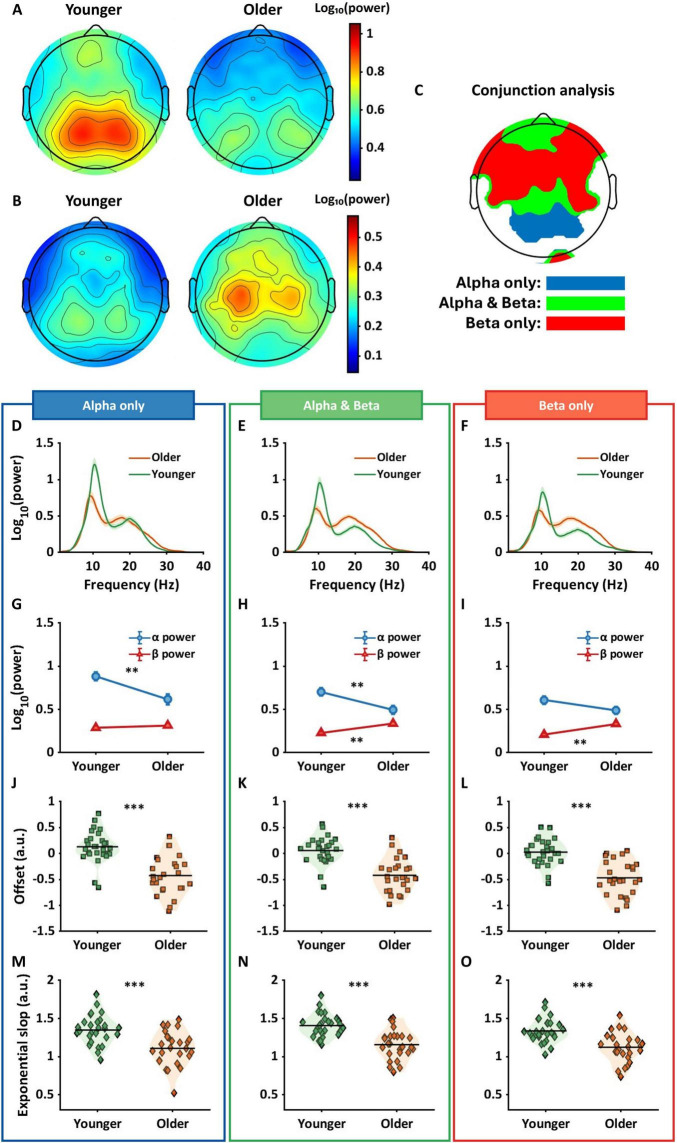
Results of two-way ANOVA and conjunction analysis in sensor space. **(A)** The topographies show alpha power for younger (left panel) and older (right panel) groups. **(B)** Topographies indicate beta power for both groups. **(C)** The conjunction analysis provided three regions that varied based on their interaction pattern. Blue regions show significant differences in alpha power only, green for the differences in both alpha and beta power, and red for the differences in beta power only. **(D–F)** The shaded line plots represent periodic power spectra ranging from 1 to 40 Hz for younger (green) and older (orange) groups. The lines and shared areas indicate means and standard errors. **(G–I)** The line plots show interactive patterns between age and frequency band. Error bars represent standard error. The significant patterns differ between the three regions with older adults showing only lower alpha power in blue regions, both lower alpha and higher beta power in green regions, and only higher beta power in red regions. **(J–O)** The violin plots show offset (squares) and exponential slope (diamonds) for each group. Horizontal lines represent means, and the shaded areas reflect the distribution of individual points. In all the regions, older adults had lower offsets and flatter slopes compared to younger adults. **p_*FDR*_ < 0.01; ***p_*FDR*_ < 0.001.

Figure 2A shows mean alpha power topographies for both groups across 128 electrodes. The left topography shows alpha power for the younger group with higher power in more posterior regions, and the right topography shows alpha power for the older group, again with higher power located more posteriorly, but attenuated relative to the younger group. Figure 2B shows topographies for beta power for each group in sensor space. In contrast to the pattern in alpha power, the older group shows an increase in beta power, primarily in electrodes over central areas, with the younger group showing attenuated beta power across large portions of the scalp. A two-way ANOVA in sensor space found significant interactions in electrodes positioned over frontal, central, parietal, and occipital regions (*p*_*FDR*_ < 0.05). Within these interaction regions, a conjunction analysis was used to identify different age by frequency band interactions.

Figure 2C represents the results from the conjunction analysis, showing three different color-coded patterns: blue for significant differences in alpha power only, green for differences in both alpha and beta power, and red for differences in beta power only. Figures 2D–F show the adjusted spectra for younger (green lines) and older (orange lines) groups within each conjunction region. Figure 2G shows mean alpha and beta power for each group. Older adults had significantly lower alpha power compared to younger adults (*p_*FDR*_* < 0.01, Hedges’ g[confidence intervals] = 0.91[0.35, 1.46]) but no significance difference in beta power (*p_*FDR*_* = 1, *g* = −0.21[−0.73, 0.32]). Figure 2H shows significantly lower alpha power (*p_*FDR*_* < 0.01, *g* = 0.84[0.29, 1.39]) and higher beta power (*p_*FDR*_* < 0.01, *g* = −1.03[−1.58, −0.46]) in older adults compared to younger adults. Figure 2I shows significantly higher beta power in older adults compared to younger adults (*p_*FDR*_* < 0.001, *g* = −1.12[−1.69, −0.55]), but no difference in alpha power.

Figures 2J–O show offset and exponential slope data for younger and older groups. Each square in Figures 2J–L and each diamond in Figures 2M–O represent an individual subjects’ offset and slope respectively. The three regions had similar age effects with older adults showing significantly lower offsets and flatter slopes compared to younger adults (all *p_*FDRs*_* < 0.001).

### Source space: age by frequency band interaction

**FIGURE 3:**
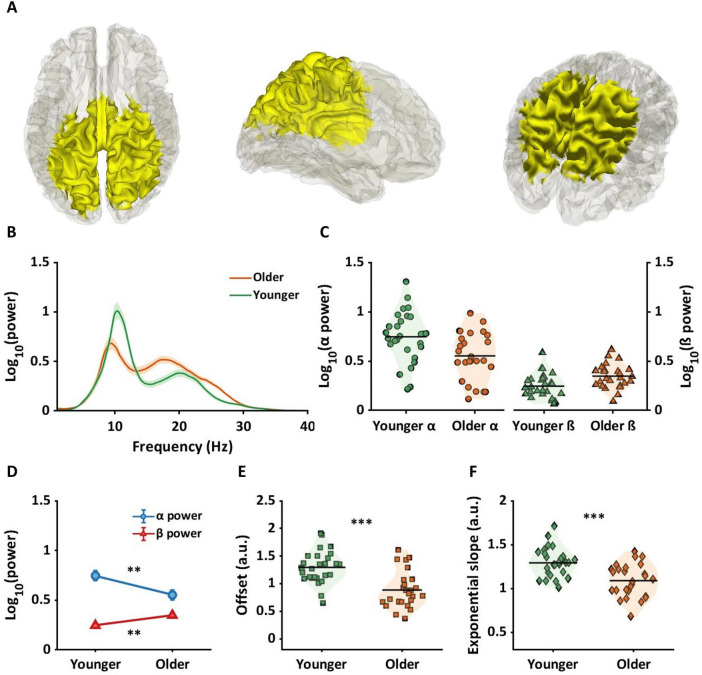
Age by frequency band interaction region and age-related alterations for alpha and beta power and offset and exponential slope in source space. **(A)** The yellow color in the brain represents the regions showing age by frequency band (alpha and beta) interactions. Below panels illustrate results for younger (green) and older (orange) adults. **(B)** The shaded line plot presents periodic power spectra ranging from 1 to 40 Hz for younger and older adults with the shaded areas indicating the standard errors. **(C)** Two violin plots in the left panel represent alpha power. The green circles indicate individual alpha power for younger adults, and the orange circles present alpha power for older adults. Horizontal lines indicate group averages. The right panel shows individual beta power for each group, represented by triangles. **(D)** The line plot shows post-hoc analysis results on alpha and beta power between age groups. Blue circles represent average alpha power, showing significantly lower alpha power in older adults. Conversely, red triangles represent beta power, indicating significantly higher beta power in older adults. **(E,F)** For aperiodic components, the violin plots illustrate offsets (squares: **E**) and exponential slopes (diamonds: **F**) between age groups, with older adults having significantly lower offsets and flatter slopes compared to younger adults. **p_*FDR*_ < 0.01; ***p_*FDR*_ < 0.001.

Figure 3A shows brain regions where significant interactions between age group and frequency band (alpha and beta) were observed. Interaction regions, highlighted in yellow, were observed in sensorimotor and parietal regions. All automated anatomical labels (AAL) regions which showed an interaction effect along with their corresponding probabilities are shown in the second column of Table 1. The highest probabilities were in precuneus, postcentral gyri, parietal gyri, and middle cingulum cortex. Figure 3B represents the averaged periodic spectrums, across all dipoles that showed an interaction, for younger adults (green line) and older adults (orange line). Figure 3C shows the average values for each subject for alpha power (circles: left panel) and beta power (triangles: right panel). The green and orange colors represent the younger and older groups, respectively. Figure 3D shows the statistical results from the post-hoc analyses. Significantly lower alpha power was found in older adults compared to younger adults (*p*_*FDR*_ < 0.01, *g* = 0.76[0.22, 1.31]). Significantly higher beta power was found in older adults compared to younger adults (*p*_*FDR*_ < 0.01, *g* = −0.82[1.37, −0.27]).

**TABLE 1:** AAL regions with significant interactions or between group differences in source space.

	Interaction regions	Alpha regions	Beta regions	Overlapping regions
AAL regions	Prob. (max = 1)	Prob. (max = 1)	Prob. (max = 1)	Prob. (max = 1)
Frontal Sup L	0.001	–	0.002	–
Frontal Sup R	0.006	–	0.010	–
Frontal Mid R	0.001	–	0.001	–
Supp Motor Area L	0.011	–	0.019	–
Supp Motor Area R	0.015	0.002	0.024	0.005
Cingulum Ant L	0.001	–	0.001	–
Cingulum Ant R	0.000	–	0.000	–
Cingulum Mid L	0.043	0.040	0.067	0.091
Cingulum Mid R	0.045	0.046	0.071	0.111
Precentral L	0.013	–	0.022	–
Precentral R	0.034	0.004	0.055	0.011
Paracentral Lobule L	0.022	0.005	0.036	0.013
Paracentral Lobule R	0.019	0.019	0.031	0.050
Postcentral L	0.041	0.003	0.069	0.008
Postcentral R	0.059	0.045	0.098	0.118
Cingulum Post L	0.014	0.022	0.002	0.005
Cingulum Post R	0.012	0.017	0.002	0.006
Thalamus L	0.008	0.001	0.012	0.002
Thalamus R	0.003	–	0.005	–
Parietal Sup L	0.048	0.059	0.038	0.049
Parietal Sup R	0.038	0.058	0.029	0.065
Precuneus L	0.080	0.120	0.050	0.103
Precuneus R	0.087	0.139	0.037	0.093
Parietal Inf L	0.042	0.020	0.060	0.029
Parietal Inf R	0.027	0.021	0.030	0.022
SupraMarginal L	0.000	–	0.000	–
SupraMarginal R	0.008	0.001	0.013	0.003
Angular L	0.012	0.012	0.004	–
Angular R	0.032	0.052	–	–
Cuneus L	0.018	0.029	–	–
Cuneus R	0.016	0.026	–	–
Calcarine L	0.007	0.011	–	–
Calcarine R	0.000	0.001	–	–
Occipital Sup L	0.025	0.041	–	–
Occipital Sup R	0.019	0.030	–	–
Occipital Mid L	0.016	0.025	–	–
Occipital Mid R	0.013	0.022	–	–
Unknown	0.162	0.131	0.209	0.218

**FIGURE 4:**
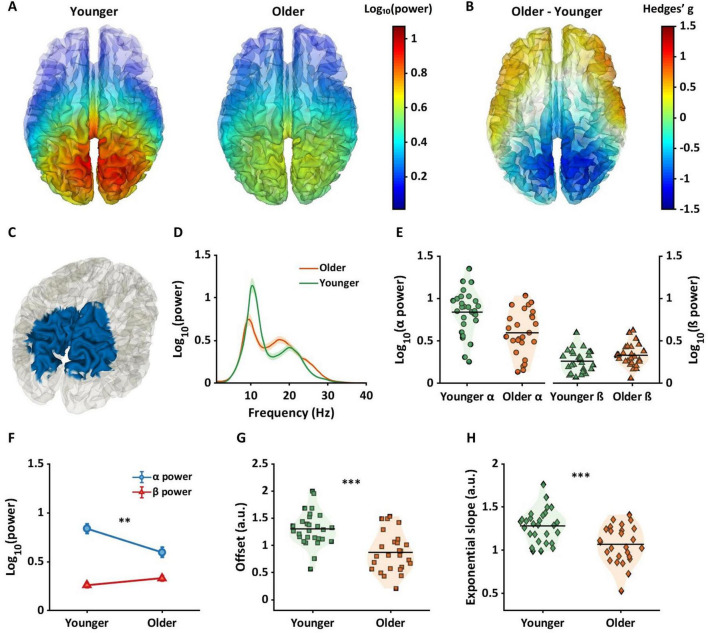
Between-group differences in alpha power, offsets, and exponential slopes in source space. **(A)** Two brain plots shows alpha power for younger (left panel) and older (right panel) across whole brain regions. **(B)** The brain plot exhibits magnitude of differences in alpha power between age groups across whole brain regions using Hedges’ g effect sizes. The warmer color indicates higher alpha power in older adults, whereas the cooler color represents lower alpha power in older adults compared to younger adults. **(C)** The blue colors are the regions where significant differences in alpha power between age groups were observed within interaction regions in Figure 3A. **(D–H)** Similar to Figures 3B–F, the plots represent the results of the blue regions **(C)** by averaging data across these regions. **(D)** Periodic power spectra ranging from 1 to 40 Hz for younger (green line) and older (orange line) adults extracted from the blue cortical region shown in panel **(C)**. **(E)** Violin plots show alpha power (left panel) and beta power (right panel) for each group extracted from the blue cortical region shown in panel **(C)**. **(F)** Older adults had lower alpha power compared to younger adults. **(G,H)** Two violin plots represent aperiodic components with older adults exhibiting lower offsets **(G)** and flatter slopes **(H)** compared to younger adults. **p_*FDR*_ < 0.01; ***p_*FDR*_ < 0.001.

**FIGURE 5:**
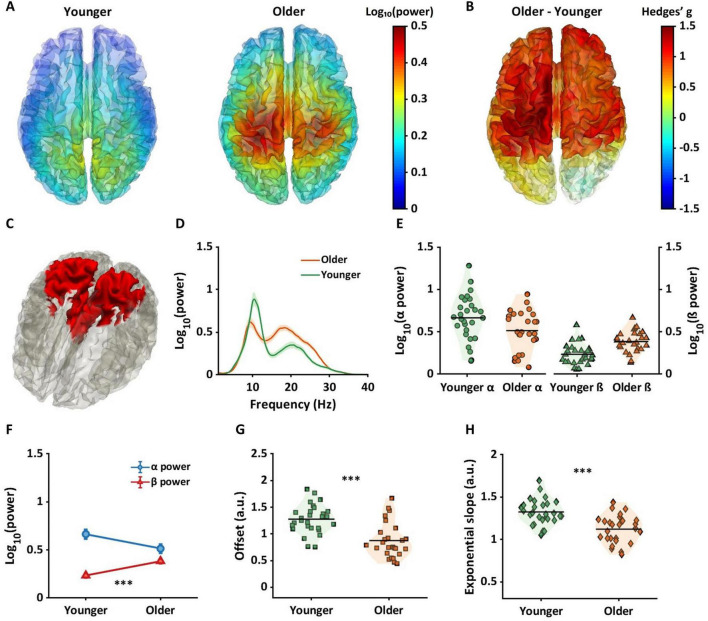
Between-group differences in beta power, offsets, and exponential slopes in source space. **(A)** Beta power in younger (left panel) and older (right panel) groups. **(B)** Hedges’ g values projected across the brain, with largest between group effects evident in the sensorimotor region. **(C)** The red color in the brain represents a binarized region where significant between group differences were found for beta power. **(D)** Periodic power spectra ranging from 1 to 40 Hz for younger (green line) and older (orange line) adults extracted from the red cortical region shown in panel **(C)**. **(E)** Violin plots show alpha power (left panel) and beta power (right panel) for each group extracted from the red cortical region shown in panel **(C)**. **(F)** Older adults had higher beta power but not alpha power compared to younger adults. For aperiodic components in this region, older adults showed lower offsets **(G)** and flatter slopes **(H)** compared to younger adults. ***p_*FDR*_ < 0.001.

**FIGURE 6:**
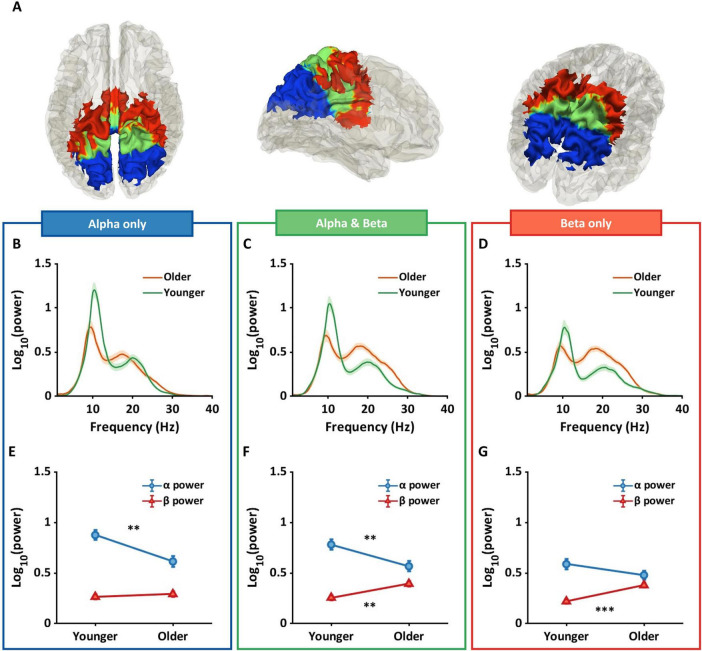
Conjunction analysis in interaction regions. The conjunction analysis was performed in source space within the interaction regions. **(A)** The analysis resulted in three distinct regions that are represented by different colors depending on their corresponding interaction patterns. **(B–D)** The shaded line plots show mean adjusted power spectral density plots for each region. **(E)** As expected, in the blue alpha only region there was a significant between group difference in alpha but not beta power. **(F)** In green, significant decreases in alpha power and increases in beta power were evident in the older group, and **(G)** the data in the red region was characterized by a significant increase in beta power. Overall, the age effect on alpha power progressively increased from parietal to sensorimotor regions, while the effect on beta power progressively decreased. **p_*FDR*_ < 0.01; ***p_*FDR*_ < 0.001.

Probabilities of brain regions where significant interactions or age effects on alpha and/or beta power were observed. The first column shows the AAL region label in the human cortex. Additional columns show the ratios of AAL regions included in the yellow areas shown in Figure 3A (interaction regions), the blue areas shown in Figure 4C (alpha), the red areas shown in Figure 5C (beta), and the green areas (overlap) shown in Figure 6A. The dash symbols indicate that the AAL regions were not included in the region. AAL, automated anatomical label; L, left hemisphere; R, right hemisphere; Sup, superior; Ant, anterior; Mid, middle; Post, posterior; Inf, inferior; Prob., probability.

Figures 3E, F show values for each subject for each aperiodic component averaged across the yellow region shown in Figure 3A. Figure 3E shows that offset values were significantly reduced in the older group compared to the younger group (*p*_*FDR*_ < 0.001, *g* = 1.30[0.71, 1.88]). Figure 3F shows that a significantly flatter slope was evident in older adults compared to the younger group (*p*_*FDR*_ < 0.001, *g* = 1.15.[0.57, 1.71]).

### Source space: between group differences in alpha power

Mean alpha power is shown across the whole brain for the younger group (Figure 4A: left) and the older group (Figure 4A: right). Figure 4B shows Hedges’ g between groups projected across all dipoles in the brain. Cooler colors indicate lower alpha power in the older group, and warmer colors represent higher alpha power in the older group. Figure 4C highlights the alpha region in blue, where significant differences in alpha power between groups were observed. Regions with the highest probabilities among the blue regions (Figure 4C) were precuneus and the superior parietal gyri (Table 1, third column). Older adults exhibited significantly lower alpha power for each dipole in the blue region.

Figures 4D–H show alpha and beta power and aperiodic components extracted from the blue region shown in Figure 4C. Figure 4D represents the periodic spectrums for younger and older groups, and Figure 4E shows individual mean alpha and beta values for each subject averaged across the blue region shown in Figure 4C. Figure 4F shows that whereas alpha power was significantly lower compared to younger adults (blue line in Figure 4F; *p*_*FDR*_ < 0.01, *g* = 0.93[0.37, 1.48]), no significant difference was evident in beta power in this region (red line in Figure 4F; *p*_*FDR*_ = 0.057, *g* = −0.52[−1.06, 0.02]).

For aperiodic components, Figure 4G shows offset values for each subject for the blue region shown in Figure 4C. Compared to younger adults (orange squares), older adults (green squares) had significantly higher offset values (*p*_*FDR*_ < 0.001, *g* = 1.26[0.68, 1.83]). Figure 4H shows exponential slope values for each subject. Compared to the younger group (green diamonds), a significantly flatter slope was found in the older group (orange diamonds) (*p*_*FDR*_ < 0.001, *g* = 1.04[0.48, 1.60]).

### Source space: between group differences in beta power

Mean beta power is shown across the whole brain in younger adults (left in Figure 5A) and older adults (right in Figure 5A). Figure 5B represents Hedges’ gs between groups projected across all dipoles. Positive numbers and warmer colors represent higher power in older adults. Figure 5C shows a binarized cortical subregion in which older adults had significantly higher beta power compared to younger adults. These regions of sensorimotor and parietal cortex were masked based on the cortical regions that showed interaction effects (see Figure 3A), and FDR corrected *p*-values. Highest probability of AAL regions included within this brain area were postcentral gyri, middle cingulum cortex, and precuneus (See fourth column in Table 1).

Figures 5D–H show the results of alpha and beta power and aperiodic components extracted from the red region in Figure 5C. Figures 5E, F show that beta power was significantly higher in older adults compared to younger adults (red line in Figure 5F; *p*_*FDR*_ < 0.001, *g* = −1.20[−1.77, −0.62]). whereas no significant difference was evident between groups in alpha power (blue line in Figure 5F; *p*_*FDR*_ = 0.03, *g* = 0.59[0.05, 1.12]),

Figure 5G shows offset values for individual subjects, averaged from dipoles shown in Figure 5C. Compared to younger adults, older adults had a significantly lower offset (Figure 5G, *p*_*FDR*_ < 0.001, *g* = 1.33[0.74, 1.91]), and flatter slope (Figure 5H, *p*_*FDR*_ < 0.001, *g* = 1.33[0.74, 1.91]).

### Source space: conjunction analysis—periodic components

Figure 6A show regions identified in the conjunction analysis: (1) alpha only (blue), (2) overlapping alpha & beta (Green), and (3) beta only (red). Figures 6B–D show periodic power spectrums for each group in each region. Green lines represent the young group, and orange lines represent the older group. Figures 6B–D show that alpha power progressively decreases from parietal (blue) to sensorimotor regions (red) in both groups, with a more prominent reduction in younger adults. In contrast, beta power is similar across regions in the older group, but is decreased in the younger group from parietal to sensorimotor regions.

Figures 6E–G show the region-specific between group differences in alpha and beta that contribute to the overall interaction effect. Blue lines represent alpha power. Red lines represent beta power. Figure 6E shows that between group differences were only evident in alpha power in parietal regions (*p*_*FDR*_ = 0.001, *g* = 0.98[0.42, 1.54]), with the red line following a horizontal trajectory and no between group difference (*p*_*FDR*_ = 1, *g* = −0.19[−0.72, 0.33]). Overlapping regions in Figure 6F showed both a significant decrease in alpha power (*p*_*FDR*_ < 0.01, *g* = 0.80[0.24, 1.34]) and a significant increase in beta power (*p*_*FDR*_ = 0.001, *g* = −1.03[−1.58, −0.46]) in the older group compared to the younger group. Finally, Figure 6G shows the opposite pattern from Figure 6E, with a significant increase in beta power in the older compared to the younger group (*p*_*FDR*_ < 0.001, *g* = −1.29[−1.87, −0.70]), but no between group difference in alpha power (*p*_*FDR*_ = 1, *g* = 0.43[−0.10, 0.96]).

### Source space: conjunction analysis—aperiodic components

**FIGURE 7:**
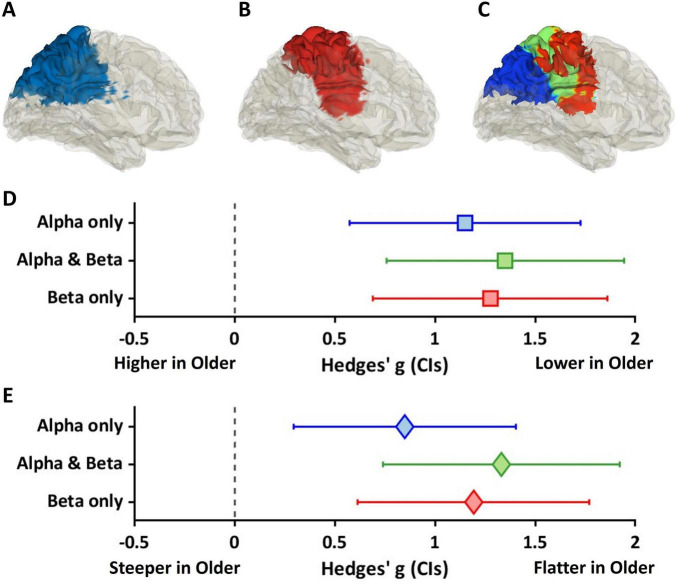
Similarity of age effects on offsets and exponential slopes across the conjunction map. **(A–C)** The left-side brain plots represent the regions where distinct patterns of age effects were observed. For example, **(A)** the blue regions show significant age effects on alpha power, **(B)** whereas the red regions exhibit significant age effects on beta power. **(C)** The conjunction map contains significant age effects on only alpha power in blue regions, both alpha and beta power in green regions, and only beta power in blue regions. **(D,E)** Two forest plots show the effects of age on aperiodic components and heterogeneity for aperiodic components between the three regions. **(D)** The forest plot exhibits Hedges’ gs for offset values between age groups, with squares representing Hedges’ g and whiskers indicating 95% confidence intervals (CIs). Each color corresponds to areas **(C)**. In the three areas, significant, positive, and large effect sizes were observed, with older adults showing lower offsets compared to younger adults. Overlapping CIs suggest similar age effects on offsets across the regions. **(E)** Similarly, another forest plot shows Hedges’ g for exponential slopes (diamonds), with significant, positive, and large effect sizes. It suggests that older adults had flatter slopes compared to younger adults. Overlapping CIs also indicates similar age effects on slopes across the regions.

Figure 7 shows results of aperiodic components for the three cortical regions identified in the conjunction analysis. Figure 7A shows sagittal views (right hemisphere) for regions of the cortex where alpha power differed between groups. Figure 7B shows regions of the cortex where beta power differed between groups. Figure 7C shows the conjunction map for alpha only (blue), alpha and beta (green), and beta only (red) regions.

Figure 7D shows Hedges’ gs values for offset values, marked by squares, with whiskers indicating 95% confidence intervals. Values are shown for regions identified in the conjunction analysis shown in Figure 7C, and are color coded accordingly. All regions showed a significant, positive, and large effect size (alpha only: blue, *p*_*FDR*_ < 0.001, *g* = 1.15[0.56, 1.72]; alpha & beta: green, *p*_*FDR*_ < 0.001, *g* = 1.35[0.76, 1.93]; beta only: red, *p*_*FDR*_ < 0.001, *g* = 1.28[0.69, 1.85]), indicating lower offsets in older adults compared to younger adults. Overlapping confidence intervals suggest that the age effect on offset was similar across regions.

Figure 7E shows Hedges’ gs between groups for exponential slope, represented by diamonds. All three regions showed significant, positive, and large effect sizes (alpha only: blue, *p*_*FDR*_ < 0.01, *g* = 0.85[0.30, 1.40]; alpha and beta: green, *p*_*FDR*_ < 0.001, *g* = 1.33[0.74, 1.91]; beta only: red *p*_*FDR*_ < 0.001, *g* = 1.19[0.61, 1.76]), revealing flatter slopes in the older group compared to the younger group. Overlapping confidence intervals suggest that the age effect on slope was similar across regions.

## Discussion

Separate studies have shown that older age is associated with changes in alpha and beta power (Polich, 1997; Vysata et al., 2012; Gómez et al., 2013; Barry and De Blasio, 2017; Cho et al., 2024). Although previous evidence points to an interaction between age and frequency band that varies across brain regions, this has not been directly tested. In the current study we address this challenge using source space analyses while also adjusting for aperiodic components (Donoghue et al., 2020). We report two novel findings. First, we found strong evidence of an age effect on aperiodic components, such that older adults had a lower offset and a flatter slope irrespective of brain region. Second, age by frequency band interactions were found in sensorimotor, parietal, and occipital regions. The pattern driving the interaction varied across regions. We found a progressive decrease in group differences in alpha power from parietal to sensorimotor regions. Beta power followed the opposite pattern, where there was a progressive increase in group differences from parietal to sensorimotor regions. We interpret our findings in the context of clinical and preclinical evidence of age effects on the cholinergic system and the Cortico-Basal Ganglia-Thalamo-Cortical (CBGTC) circuit.

EEG signals contain unique background power spectra that can be influenced by factors including age and neurologic diseases (Pani et al., 2022). With healthy aging, previous studies have reported a downward shift in the background power spectra in older adults (Donoghue et al., 2020; Merkin et al., 2023; Tröndle et al., 2023). This shift results in a lower offset and a flatter slope, potentially reflecting lower cortical neuronal spiking and decreased inhibitory activity. Our observations are consistent with this finding. We extend the literature by showing lower offsets and flatter slopes in older adults across large portions of the scalp and cortex. Offset and slope data follow unimodal distributions in each age group as shown in Supplementary Figure 2. Aperiodic components can attenuate age-related differences in alpha power but unmask age-related differences in beta power (Merkin et al., 2023).

Aperiodic components are distinct from periodic components in that they are not altered by acute changes in neural activity, such as during visual processing or performing a motor task. Instead, changes in aperiodic components appear to be influenced by much slower moving processes such as neurological diseases (Pani et al., 2022) and age-related degeneration (Donoghue et al., 2020; Merkin et al., 2023; Tröndle et al., 2023). This raises the question as to whether the periodic and aperiodic components represent distinct neural processes. Several lines of evidence suggest that this is indeed the case. Wan et al. (2019) investigated resting state alpha reactivity in healthy older adults, which measures the change in alpha power when transitioning from an eyes-closed to an eyes-open condition. Figure 3A in their paper shows the unadjusted power spectrum averaged across subjects. The periodic alpha reduction with eyes open is displayed above the aperiodic power spectrum. In contrast, the aperiodic power spectrum does not change. Given that periodic power changes could be region specific and that aperiodic components could alter how the raw data are interpreted, it was important to assess and control for aperiodic components separately within distinct brain regions. Aperiodic components are known to originate from cortical neuronal spiking (Manning et al., 2009) and a ratio between excitatory and inhibitory activity in the neocortex (Gao et al., 2017), whereas the generation of periodic alpha and beta power is associated with the cholinergic circuit (Eggermann et al., 2014) and the CBGTC circuit (Reis et al., 2019) respectively. Given the broadband effect of age on aperiodic components and the region-specific effect of age on periodic components, our findings point toward distinct neural circuits underlying alpha and beta power, with between group differences emerging in opposite directions within these frequency bands.

### Lower alpha power in older adults in occipital and parietal but not sensorimotor regions

Our finding of an age-related reduction in alpha power in occipital and parietal regions aligns with previous studies (Polich, 1997; Babiloni et al., 2006; Rossini et al., 2007; Vysata et al., 2012; Gómez et al., 2013; Barry and De Blasio, 2017; Scally et al., 2018; Jabès et al., 2021; Doval et al., 2023; Merkin et al., 2023; Tröndle et al., 2023; Cho et al., 2024). In sensor space in posterior regions, an age-related decrease in alpha power has been found using between group (Barry and De Blasio, 2017; Tröndle et al., 2023) and correlation (Polich, 1997; Gómez et al., 2013) analyses. Evidence from source space analyses shows similar patterns of age-related reductions in alpha power in parietal and occipital regions (Babiloni et al., 2006; Cho et al., 2024), and this pattern holds after correction for aperiodic components in most (Tröndle et al., 2023; Park et al., 2024), but not all studies (Merkin et al., 2023).

Our results differed from the Merkin et al. (2023) finding for several potential reasons. First, removing aperiodic components may reduce the age effects on alpha power, as the alpha power in older adults shifted downward prior to the adjustment. This idea is supported by Tröndle et al. (2023), who demonstrated that the effect size (Cohen’s d) of age decreased from approximately 0.6 to 0.4 following the aperiodic correction. Previous studies also suggest that aperiodic components might overestimate the age effects on alpha power (Donoghue et al., 2020; Merkin et al., 2023; Tröndle et al., 2023). Second, participant heterogeneity could be a factor, as Merkin et al. (2023) combined datasets from six studies, using two different EEG systems. Tröndle et al. (2023) also merged two independent datasets and found no significant age effects on alpha power. However, when running statistics for each data set separately, effects did emerge in one data set but not the other (Tröndle et al., 2023). Combining datasets may therefore introduce heterogeneity. Third, Merkin et al. (2023) calculated power at the peak alpha frequency, whereas we averaged power from 8 to 13 Hz. Despite these differences, what is clear from the evolving literature is that age effects on aperiodic components appear to be robust and consistent across different studies. Indeed, relative to periodic power, aperiodic processes maybe more sensitive to aging, and less sensitive to differences in methodology between studies.

Animal studies have provided important insight into the potential causal links between cholinergic function in the basal forebrain and posterior alpha oscillations (Gallagher and Colombo, 1995; Ricceri et al., 2004; Eggermann et al., 2014). Anatomical tracing studies have shown that cholinergic projections of the basal forebrain are connected to cortical interneurons (Sokhadze et al., 2022) via the thalamus (Woolf and Butcher, 1986; Hallanger et al., 1987; Steriade et al., 1987; Jourdain et al., 1989; Sokhadze et al., 2019), a key structure thought to underlie the generation of posterior alpha oscillations (Hughes and Crunelli, 2005; Lõrincz et al., 2009; Nestvogel and McCormick, 2022). Indeed, impaired cholinergic input to the basal forebrain decreases alpha power (Gallagher and Colombo, 1995; Ricceri et al., 2004) and inhibiting visual thalamus eliminates posterior alpha oscillations in visual cortex (Nestvogel and McCormick, 2022). Cholinergic projections degenerate with aging, showing atrophy (Ballinger et al., 2016; Choi et al., 2022) and lower cardiorespiratory fitness is associated with reduced resting state functional connectivity between the nucleus basalis of Meynert and right middle frontal gyrus in older adults (Won et al., 2023). A recent human study using EEG, functional and diffusion MRI, revealed that an accumulation of leukoaraiosis, which reduces connectivity between the basal forebrain and neocortex, was associated with an attenuated change in posterior alpha power when transitioning from an eyes open to an eyes closed state, referred as to alpha reactivity (Wan et al., 2019). Reduced alpha reactivity in posterior regions is a hallmark symptom of Lewy Body dementia (Schumacher et al., 2020) which is an age-related neural disease that has been associated with deficits in the cholinergic circuit. Hence, in addition to an age-effect on peak alpha frequency, there is growing evidence that alpha power over posterior regions of occipital and parietal cortex has the potential to serve as an indirect readout of cholinergic system function.

### Higher beta power in older adults in sensorimotor and parietal but not occipital regions

Relative to the younger group, beta power was increased in the older group in parietal and sensorimotor cortex. Prior studies have reported an age related increase in beta power (Barry and De Blasio, 2017; Heinrichs-Graham et al., 2018; Rempe et al., 2023; Cho et al., 2024), a decrease in beta power (Vysata et al., 2012), or no age-related change (Babiloni et al., 2006; Jabès et al., 2021). While the majority of studies converge on an age-related increase in beta power in parietal and sensorimotor regions, heterogeneity in the data is clear and may be attributed to factors other than age. For instance, factors such as education (Harmony et al., 1990), sex (van Putten et al., 2018), cognitive reserve (Balart-Sánchez et al., 2021), and fitness level (Lardon and Polich, 1996) may also influence beta power independently of age, but are rarely controlled for. Future studies that explore aging effects would be strengthened by considering and/or controlling for these other factors. Another potential source of heterogeneity may come from how beta power is calculated and whether or not aperiodic components are controlled for. Absolute measures of beta power do not consider aperiodic changes such that shifts in the slope and offset of the power spectra are conflated into the interpretation of the periodic component. In contrast, relative power can help control for aperiodic components by normalizing the entire power spectra across frequencies. Indeed, consistent with our finding, studies that calculated relative beta power in sensorimotor cortex have reported an age-related increase (Vysata et al., 2012; Gómez et al., 2013; Jabès et al., 2021; Rempe et al., 2022, 2023; Doval et al., 2023). Hence, calculating relative power or isolating aperiodic components should also be considered to accurately assess periodic beta power.

Age-related increases in sensorimotor beta power are associated with age-related movement slowing (Heinrichs-Graham et al., 2016, 2018). Heinrichs-Graham et al. (2016) used MEG to demonstrate that absolute levels of beta were higher in older adults at rest, and that slower movement times were associated with a larger decrease in power that must occur in order for older adults to reach an absolute movement threshold. Additional evidence in support of this association comes from studies in healthy adults, which showed that individuals with slower motor performance exhibited higher sensorimotor beta power during rest (Gilbertson et al., 2005; Rassi et al., 2023). Moreover, patients with Parkinson’s disease (PD), a condition characterized by slower movement, have reported increased sensorimotor beta power (Pollok et al., 2012; Gimenez-Aparisi et al., 2023; Karekal et al., 2023). An increase in sensorimotor beta power may be attributed to neurodegeneration in the CBGTC circuit (Simonyan, 2019; Barone and Rossiter, 2021; Navarro-López et al., 2021), a key regulator of beta oscillations in sensorimotor cortex (Bhatt et al., 2016; Sherman et al., 2016; Chikermane et al., 2024). Causality in human studies has not been established, but animal and modeling studies have shown that dysfunction in the CBGTC circuit exacerbates PD symptoms (Galvan et al., 2015; McGregor and Nelson, 2019; Navarro-López et al., 2021), and is associated with increases in sensorimotor beta power (Reis et al., 2019). The reverse is also true such that deep brain stimulation targeting the subthalamic nucleus, a key part of CBGTC circuit, alleviates PD-related motor symptoms and attenuates sensorimotor beta power (Abbasi et al., 2018; Luoma et al., 2018). Hence, one interpretation of our finding is that even in healthy aging, deficits in structure or function of the CBGTC circuit (Corregidor-Sánchez et al., 2020) may underlie the emergence of increased sensorimotor beta power. Longitudinal studies that assess both degeneration in the CBGTC circuit and beta power will be necessary to move the field toward a causal understanding of this relationship.

The current study used a cross sectional between group design. However, given that age-related increases in beta power appear to be progressive over time (Polich, 1997; Vysata et al., 2012; Gómez et al., 2013; Rempe et al., 2022, 2023), longitudinal lifespan studies that assess EEG measures in combination with measures of CBGTC circuit structure and function remain critical. Furthermore, our sample included more females than males. A previous study reported that female adults tend to show higher broadband power relative to males (Cave and Barry, 2021), which may have contributed to the effects of age on alpha and beta power in the current study. However, since the Cave and Barry study did not control for aperiodic components, the sex effect on periodic components remains unclear. Nevertheless, our findings may not generalize well to males, and we encourage future appropriately powered studies to explore the interacting effects of age and sex on periodic alpha and beta power. In addition, the lack of clinical and demographic information related to fitness levels, education, cognition, and motor skills is a limitation of this study, as these factors are known to influence age-related changes in alpha and beta power (Heinrichs-Graham et al., 2018; Gramkow et al., 2020; Cesnaite et al., 2023). Given conflicting previous findings on the associations between age and alpha power and the within group variability within each group in the current study (see Figures 3C, 4E, 5E), we note that despite strong group-differences, alpha power may not be the most reliable biomarker of age. Indeed, factors such as physical activity (Lardon and Polich, 1996), cardiovascular health (Vecchio et al., 2012), or cognitive/neural reserve (Balart-Sánchez et al., 2021) may influence neural activity, making a single biomarker for aging challenging. Future studies will be necessary to determine the value of alpha power, alone or in combination with other measures, as a reliable biomarker for age. We also note that the focus on alpha and beta power in the current study prevented new information on age effects on theta and gamma power. Given the different roles between cortical alpha/beta (feedback or top-down process) and gamma power (feedforward or bottom-up) (Mejias et al., 2016; Michalareas et al., 2016), testing the interaction effect between age and these frequencies power may be an interesting topic to better understand age-related degeneration in cortical neural signals.

Taken together, this study bridges a literature gap by examining aperiodic and periodic components in sensor and source space and adjusting for aperiodic components. We note two important observations. First, older adults had a lower offset and flatter slope across large portions of sensor and source space. Second, age and frequency band interactions were seen in sensorimotor, parietal, and occipital regions, with unique patterns driving the interaction across regions. Age-related alterations in alpha power progressively decreased, and the alteration in beta power progressively increased from parietal to sensorimotor regions.

## Data Availability

The original contributions presented in this study are included in this article/Supplementary material, further inquiries can be directed to the corresponding author.
