# Supplementary material for: Age-related changes in neural oscillations vary as a function of brain region and frequency band
Source: Front Aging Neurosci. 2025 Feb 18;17:1488811. doi: 10.3389/fnagi.2025.1488811 (PMC11876397; doi:10.3389/fnagi.2025.1488811)
Supplement: Supplementary file 1 [file Table_1.docx]

**Age-related changes in neural oscillations vary as a function of brain region and frequency band**

Jinhan Park^1^, Rachel L.M. Ho^1^, Wei-en Wang^1^, Shannon Y. Chiu^2^, Young Seon Shin^1^, and Stephen A. Coombes^1, 3, *^

^1^ Laboratory for Rehabilitation Neuroscience, Department of Applied Physiology and Kinesiology, University of Florida, Gainesville, FL 32611, USA

^2^ Department of Neurology, Mayo Clinic, Scottsdale, Arizona, USA

^3^ Department of Biomedical Engineering, University of Florida, Gainesville, FL 32611, USA

**Supplementary materials**

Contents

[Table S1. Parameters for the resting-state EEG analysis 2](#_Toc188359731)

[Figure S1. The accuracy of model fits resulting from the FOOOF algorithm 4](#_Toc188359732)

[Figure S2. Offset and slope values for younger and older groups 5](#_Toc188359733)

## Table S1. Parameters for the resting-state EEG analysis

| Preprocessing | Sub-category | Function | Parameter |
| --- | --- | --- | --- |
| Data processing | Down sampling | pop_resample | 256 Hz |
|  | Segmenting middle data | pop_select | 60-540 seconds |
|  | Channel location | pop_chanedit  pop_chancenter | polhemus  hearadian = 85 |
|  | Band pass filter | pop_eegfiltnew | Low cutoff = 1 Hz  High cutoff = 100 Hz  Order = 6760 |
|  | Remove line noise | pop_cleanline | Bandwidth = 2  Line frequency = 60 and 120 |
|  | Remove bad channel’s signals | pop_clean_rawdata | LineNoiseCriterion = 4 |
|  | Interpolation | pop_interp | Spherical method |
|  | Re-reference | pop_reref |  |
|  |  |  |  |
| Independent component analysis | AMICA | runamica15 | Number of rejections = 15  Number of standard deviations for rejection = 3  Iteration interval between rejection for rejections = 1 |
|  | Remove non-brain ICs | pop_iclabel  pop_icflag  pop_subcomp | Brain = 0%  Muscle = 80%  Eye = 50%  Heart = 0%  Line Noise = 0%  Channel Noise = 0%  Other = 0% |
|  |  |  |  |
| Artefact removal | Remove bad data points | pop_clean_rawdata | BurstCriterion = 20  WindowCriterion = 0.25  WindowCriterionTolerances = -Inf-7 |
|  | Segment epochs | eeg_regepochs | 6 seconds (-2-4 seconds) |
|  | Automatic epoch rejection | pop_eegthresh  pop_jointprob  pop_rejkurt | Outlier value = ± 100 µV²  Joint probability = 5  Kurtosis = 5 |
|  |  |  |  |
| Source localization | Dipole fitting | pop_dipfit_settings | Head template = BEM  Coordinates = MNI space  Channel = 10-5 systems  Co-registration = [1 -20 -8 -0.20944 0 -1.5708 1.6 1.6 1.8] |
|  | Remove dipole | pop_multifit | Residual variance = 100%  Removing dipoles located in outside of the brain |
|  | eLORETA | ft_read_headshape | Building source map based on “cortex_20484.surf.gii”. |
|  |  | Inverse solution | BEM  cortex_20484.surf.gii  eloreta |
| Parameterizing neural components | FOOOF | fooof_check_settings | peak_width_limits = [0.5, 12]  max_n_peaks = inf  min_peak_height = 0  peak_threshold = 2.5  aperiodic_mode = fixed  verbose = true |
|  |  | fooof | Frequency ranging from 1 to 40 Hz  Power spectrum density (PSD) in sensor or source space  Fooof check settings |
| Alpha & Beta power | Alpha power | nanmean | Averaging PSD within 7-13 Hz |
|  | Beta power | nanmean | Averaging PSD within 13.1-30 Hz |

## Figure S1. The accuracy of model fits resulting from the FOOOF algorithm


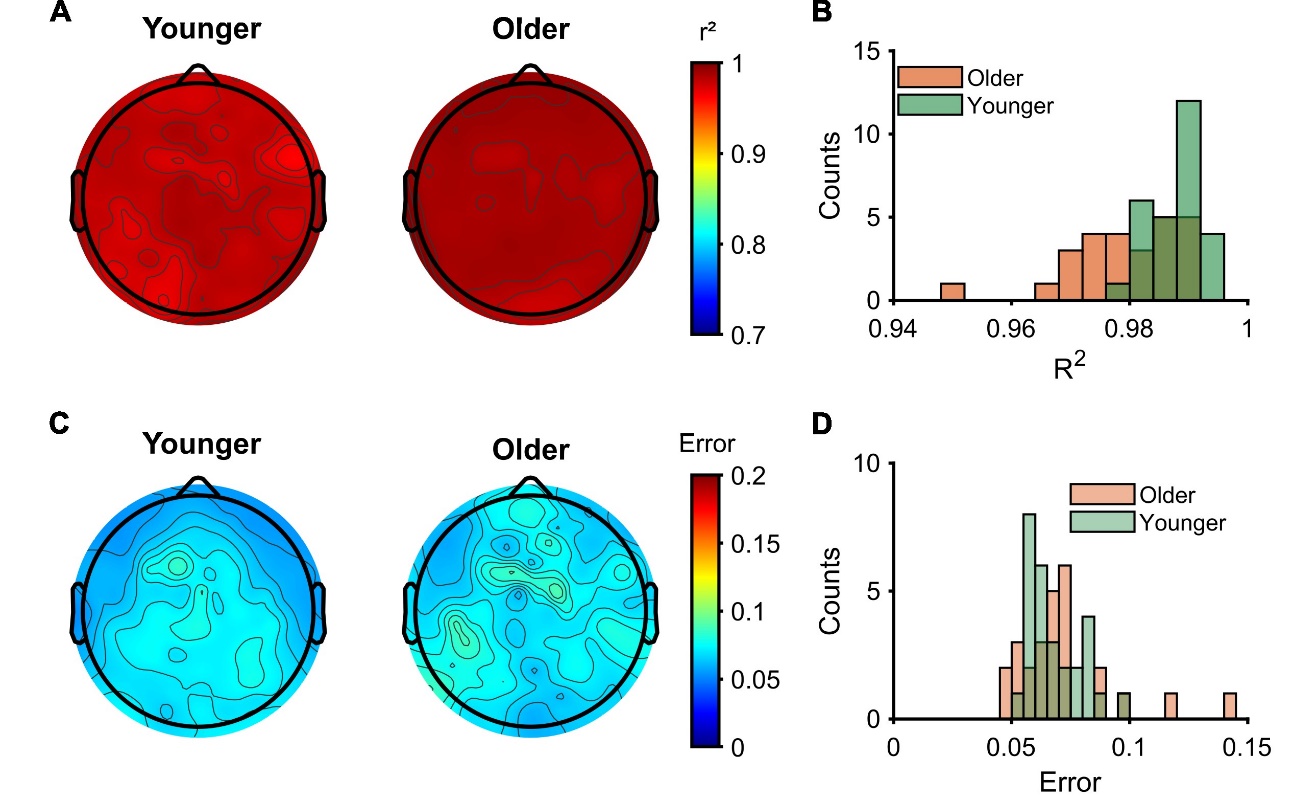


*Note.* Figure S1 illustrates the accuracy of FOOOF model fits in sensor space. **A**. The topographies show the group-averaged r-squared values for the younger (left panel) and older (right panel) groups, meaning the magnitude to explain original power spectra by the model fitted power spectra. **B**. The histogram represents the distribution of the r-squared values averaged across 128 channels for younger (green) and older (orange) groups. Both figures exhibited that both groups had greater r-squared values than 0.94, suggesting that the model fits accurately explained the original power spectra. **C-D**. The figures illustrate error values across 128 channels as similar as Figures **A-B**. The value is the mean squared error between the original power spectra and the model fit, showing relatively low error values for both groups. Given these findings, the possibility of bias or noise from FOOOF model fits is minimal.

## Figure S2. Offset and slope values for younger and older groups


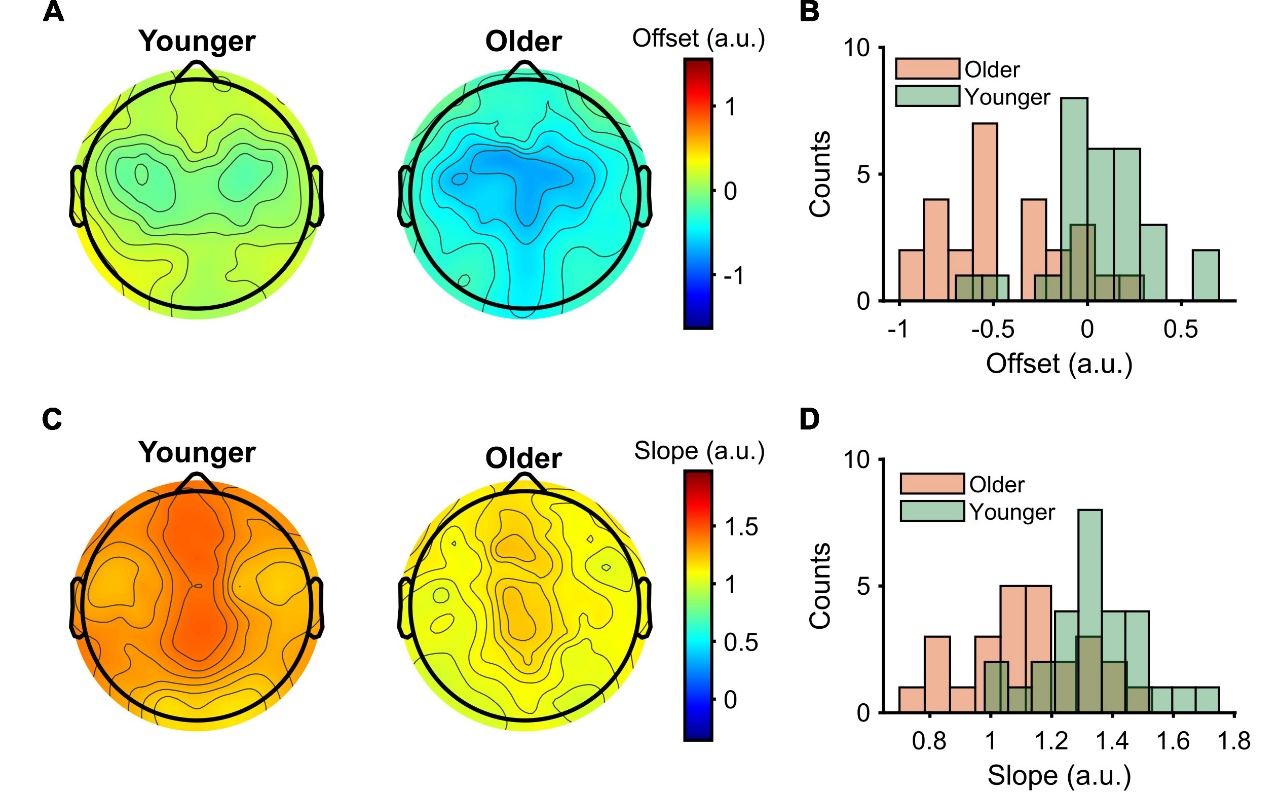


*Note.* Figure S2 illustrates offset and slope values for both groups in sensor level. **A**. The topographies show offset values across all electrodes for the younger (left panel) and older (right panel) groups. **B.** The histogram represents the distributions for the younger (green) and older (orange) groups. The offset values used in the histogram were averaged across 128 electrodes. From our perspective, these distributions appear to be unimodal, suggesting a lower likelihood of potential subgroups. Similarly, **C and D** display exponential slope values. **D** shows histograms for both groups, which also follow unimodal distributions. These results suggest that offset and slope values are well explained by age groups.
